# Resilience and Physical and Mental Well-Being in Adults with and Without HIV

**DOI:** 10.1007/s10461-017-1980-6

**Published:** 2017-11-20

**Authors:** Jennifer A. McGowan, James Brown, Fiona C. Lampe, Marc Lipman, Colette Smith, Alison Rodger

**Affiliations:** 1 Department of Epidemiology & Public Health, University College London, 1–19 Torrington Place, London, WC1E 6BT UK; 2 Department of Respiratory Medicine, Royal Free London NHS Foundation Trust, London, UK; 3 Research Department of Infection and Population Health, University College London, London, UK; 4 UCL Respiratory, Division of Medicine, University College London, London, UK

**Keywords:** Resilience, Age, Time with HIV, Mental health, QoL

## Abstract

Resilience has been related to improved physical and mental health, and is thought to improve with age. No studies have explored the relationship between resilience, ageing with HIV, and well-being. A cross sectional observational study performed on UK HIV positive (N = 195) and HIV negative adults (N = 130). Associations of both age and ‘time diagnosed with HIV’ with resilience (RS-14) were assessed, and the association of resilience with depression, anxiety symptoms (PHQ-9 and GAD-7), and problems with activities of daily living (ADLs) (Euroqol 5D-3L). In a multivariable model, HIV status overall was not related to resilience. However, longer time diagnosed with HIV was related to lower resilience, and older age showed a non-significant trend towards higher resilience. In adults with HIV, high resilience was related to a lower prevalence of depression, anxiety, and problems with ADLs. It may be necessary to consider resilience when exploring the well-being of adults ageing with HIV.

## Introduction

Resilience is a multidimensional concept which seeks to explain how some individuals can attain, maintain, or regain well-being in the face of hardship [1]. It encompasses an individual’s current coping resources in response to stressors, and their ability to effectively adapt these resources to manage new stressful situations [2–6]. Resilience can be broken down into five intrinsic characteristics: purpose/meaning in life [7–9], perseverance [10], equanimity (balance, composure) [10, 11], self-reliance [1, 7, 8, 10, 12] and ‘authenticity’ (self-acceptance, autonomy) [7]. Often, definitions of resilience also rely on the premise that the individual has experienced stressful life events before and can utilise these experiences to effectively adapt their behaviours [13–15].

Higher levels of resilience have been consistently linked to improved mental and physical health in the general population [16, 17]. This has been found in relation to chronic illness, health-care behaviours, and disability. Large longitudinal studies have also provided evidence that resilience may be a protective factor against the development of disability, chronic illness, depression, or low Health-related Quality of Life (HrQoL) [18–20], suggesting a possible causal relationship between high levels of resilience and overall well-being. Furthermore, many resilience studies focus on chronically ill populations, and have found evidence that it is possible to develop or maintain resilience despite poor physical health [19, 21, 22]. This implies that resilience can be used to improve well-being within chronically ill populations.

In the general population, older adults are consistently found to have high levels of resilience [20, 23, 24], which is thought to be due to accrued experiences with adversity—such as bereavement, physical changes, and adaptive life roles [25]. Within people with HIV (PWH) it is possible that age, and increased time with diagnosed HIV, allow adults to learn and hone their coping strategies such that resilience increases. However it is also possible that illness-related stress or poor mental health impairs this population’s ability to develop resilience in a manner similar to that of the toxic stress model [26], which states that prolonged stress and illness may inhibit the time and resources necessary for positive mental development. Research is required to identify the relationship between resilience and well-being in PWH by age and time diagnosed with HIV.

UNAIDS estimate that in high income countries 33.3% of PWH are over the age of 50 [27]. In the UK specifically, the number of older adults with HIV has tripled in the past decade [28], making older adults with HIV an important population for researchers and clinicians to understand. A recent multicentre cross-sectional UK study (ASTRA) reported that longer time diagnosed with HIV was associated with poorer self-reported mental and physical health [29]. However, only two previous quantitative studies were identified in relation to HIV and resilience. The first compared resilience in people with and without HIV (N = 60) using the CD-RISC scale [30]. This study found some evidence of lower resilience in PWH when compared to the HIV-negative group (mean score = 27.0 vs. 31.0, p = 0.06) [30]. The second study included 138 women with HIV in the USA, and found that high resilience was associated with increased antiretroviral therapy (ART) adherence and a greater likelihood of undetectable HIV load, when controlled for age, race, income, substance use, and depressive symptoms [31]. Neither study explored the association between age and resilience in PWH, the association between resilience and time diagnosed with HIV, or the associations between resilience and physical or mental health in this population. As such, very little is known about the prevalence or importance of resilience in PWH.

The primary aims of this study were to (a) assess the association between HIV status and resilience, (b) assess the association of age with resilience in adults with and without HIV, (c) assess the association of ‘time with diagnosed HIV’ with resilience in adults with HIV and (d) assess the association of resilience with depression, anxiety, and problems with activities of daily living (ADLs) in adults with and without HIV.

## Methods

The study aimed to recruit a group of PWH and a group of HIV-negative individuals. Individuals with diagnosed HIV attending the ambulatory care service at the Ian Charleson Centre (ICC) HIV clinic between March 2015 and August 2015, and individuals with or without diagnosed HIV attending the Marlborough Sexual Health Clinic over the same period, were approached and invited to participate in the study. Both clinics are situated at the Royal Free London NHS Foundation Trust, and provide HIV testing. The Marlborough clinic has high rates of HIV testing (99% offer and 86% uptake). Participants recruited from the Marlborough Clinic therefore reflect a population that are sexually active, but unlikely to have undiagnosed HIV infection.

Recruitment was done in specific clinical sessions within which all eligible patients were approached. All individuals who agreed to participate in the study gave written informed consent and completed a self-administered confidential pen-and-paper questionnaire. Participants in the Marlborough Clinic were asked to self identify their HIV status at enrolment (all reported they were HIV negative) and given the HIV positive, or negative, questionnaire as appropriate. The two versions of the questionnaire included identical items on a range of demographic, health, and lifestyle issues; the HIV-positive version also included an additional question relating to date of HIV diagnosis. The research protocol and all versions of the study documents (information sheet, consent form and questionnaire) were approved by the London, Camden & Islington Research Ethics Committee (ref 14.LO.1646).

Patients were eligible if they: (a) were over 18 years of age, (b) had sufficient English fluency to be able to understand the information sheet, questionnaire and provide informed consent, and (c) were not too ill or distressed to participate, as judged by the clinic staff. In addition, HIV-negative participants were further required to be aged 30 or over, to ensure a more comparable age distribution to that of the HIV positive population, as respondents from this clinic were found to be significantly younger than those from the HIV clinic in early data collection.

During the study period, 439 participants were invited to take part in the study, of which 327 completed the questionnaire (overall 74.5% response rate; 76% for HIV positive and 73% for HIV negative participants). No comparison data was available on the difference in demographics between respondents and non-respondents at either clinic.

### Symptom Measures

The RS-14 [2] was used to measure resilience. This uses a 14-item, 7-point Likert scale. The Resilience Scale (RS) [2] was specifically developed to measure resilience in older adults [25], and a recent systematic review concluded that the scale had ‘psychometric rigor’, as defined via an analysis of content validity, internal consistency, criterion validity, construct validity, reproducibility, responsiveness, floor and ceiling effects and interpretability [32]. As such it was chosen for this study. The RS-14 scores are summed to create a resilience reading (range 14–98). A summated score of ≥ 82 is taken to indicate high resilience; 74-81 indicates moderate resilience; and ≤ 73 indicates low resilience [10]. These are the accepted groupings for measuring resilience using this scale [10]. For the purpose of logistic regression analysis participants reporting ‘high’ (scores ≥ 82) were compared to those reporting ‘moderate—low’ resilience scores (scores < 82).

Mental health was assessed using clinically validated questionnaire measures of depression and anxiety. Specifically, depression and anxiety symptoms were determined via self-report, using the PHQ-9 [33] and GAD-7 scales [34] respectively. These scales are validated diagnostic instruments, based on the DSM-IV criteria for depression [33] and generalised anxiety disorder (GAD) [34]. Both scales inquire about the frequency of occurrence of specific symptoms in the past 2 weeks: the presence of depression and anxiety are defined according to standard algorithms based on a total score of 10 or more in each case. Binary variables were used as these were considered to have the most clinical relevance.

Difficulty with ADLs was identified using the EQ-5D [35], a five-item quality of life instrument. This instrument asks participants to report their ‘status of health today’ in relation to Mobility, Self-care, ‘Usual activities’ (e.g. work, study, housework, family, or leisure activities), Pain/discomfort and Anxiety/Depression. Response options comprise a five-point Likert scale. In this analysis, measures of difficulty with ADLs were derived using the first three domains only. An overall measure of problems with physical function was used, where participants who reported problems (responses of ‘slight problems’, ‘moderate problems’, ‘severe problems’ or ‘unable to perform each activity’) in at least one of the three domains were classified as having a physical function problem. In addition, the three individual domains were also considered separately.

Other demographic factors included in the analysis were age group (< 40, 40–49, ≥ 50 years), self-reported gender/sexuality (MSM, heterosexual men, women) and country of birth (UK-born or other). The age groups were chosen to coincide with the ‘older age’ group of ≥ 50 years which is commonly used in the HIV literature, and coincides with the age from which well-being is thought to improve in the general population [36], and to allow for the age restrictions imposed by necessity on the HIV negative participants.

### Statistical Analysis

The distribution of the resilience score was examined among HIV-positive and HIV-negative samples, and means were compared using an unpaired *t* test. The association of HIV-status with high resilience (score ≥ 82) was assessed using logistic regression analysis, adjusted for age group, gender/sexual orientation and country of birth. Chi squared test for trend was used to assess the association of age group with resilience separately among HIV-positive and HIV-negative groups. Logistic regression was used to assess the adjusted associations of age group, gender/sexual orientation, and country of birth with high resilience (score ≥ 82) separately among the HIV-positive and HIV-negative groups. In addition, time since HIV diagnosis (< 5 years; 5–10 years; 10–20 years and > 20 years) was included in the model for the HIV-positive group. Age and time since diagnosis of HIV (where appropriate) were fitted as categorical variables in logistic regression models in order to allow for non-linear patterns of association. Tests for linear trend across age and time with diagnosed HIV were also performed to identify linear differences in resilience and well-being across the HIV positive and negative groups. However, it was considered important to categorise age into groups, rather than as a continuous variable, to allow the exploration of non-linear age differences. Age group, gender/sexual orientation, country of birth and time with diagnosed HIV were chosen as independent factors to include in the multivariable logistic models, because these factors were considered potential confounders for which any causal association with resilience was likely to operate in one direction only.

The prevalence of depression, anxiety, and problems with ADLs (as defined above) were compared between HIV-positive and HIV-negative groups. The associations of resilience (categorised as low (RS-14 score ≤ 73), average (74–81), and high (≥ 82)) with depression, anxiety, and problems with ADLs, were assessed using Chi squared tests for trend for the HIV-positive and HIV-negative groups separately. Logistic regression analysis was used to identify the association of age, gender/sexual orientation, country of birth, time with diagnosed HIV (for the HIV-positive group only) and resilience (categorised in three groups as defined above)with each of the three well-being measures (depression, anxiety, problems with ADLs) using two stages of analysis.

The initial analyses (Step 1) models included as independent variables: age group, gender/sexuality, country of birth, and time since diagnosis of HIV (for the HIV-positive group only). The analyses were then rerun (Step 2) with the inclusion of resilience as an additional independent variable (low-average (< 82); high (≥ 82)) in order to assess the extent to which resilience might ‘explain’ any associations of age and time with diagnosed HIV with depression, anxiety, or problems with ADLs.

## Results

### Subject Characteristics

The final HIV positive study population included 195 participants. The HIV negative sample included 126 participants. In the HIV positive group, the mean age was 48 years (SD = 10.5, range 19–82 years). The mean age for the HIV negative population was 42 (SD = 11.2, range 19–81) (Table 1). As such, the HIV positive population was significantly older than the HIV negative population (p < 0.001), despite the recruitment measures used.

**Table 1:** Demographic and health-related factors in the HIV- positive and HIV-negative samples (n & percentage of group)

Characteristics	HIV positive population	HIV negative population
	(N = 195)	(N = 126)
Age (years)^a^		
Mean (SD)	48 (10.5)	42 (11.2)
< 40	40 (20.5%)	68 (54.0%)
40–50	72 (36.9%)	31 (24.6%)
50–60	61 (31.3%)	17 (13.5%)
60 +	22 (11.3%)	10 (7.9%)
Gender/sexuality^a^		
MSM	131 (66.5%)	30 (24.2%)
Het. Male	27 (13.7%)	58 (46.8%)
Female	39 (19.8%)	36 (29.0%)
Ethnicity		
Born in the UK	121 (61.7%)	72 (56.7%)
White British	106 (54.1%)	49 (38.9%)
Black African	32 (16.3%)	8 (6.3%)
Work status^a^		
Employed	114 (58.2%)	102 (81.6%)
Unemployed	25 (12.8%)	5 (4.0%)
Sick/disabled	28 (14.2%)	5 (4.0%)
Retired	18 (9.2%)	4 (3.2%)
Other	11 (5.6%)	9 (7.2%)
Highest education level		
University or above	106 (54.4%)	74 (60.7%)
A levels	31 (15.9%)	18 (14.8%)
GCSE/O-level	28 (14.4%)	11 (9.0%)
None	24 (12.3%)	14 (11.5%)
Money for basic needs		
Always	114 (57.9%)	83 (66.4%)
Mostly	49 (24.9%)	31 (24.8)
Sometimes	18 (9.1%)	8 (6.4%)
Never	16 (8.1%)	3 (2.4%)
Smoking status		
Current	58 (30.2%)	45 (36.0%)
Ex-smoker	54 (28.1%)	23 (18.4%)
Non-smoker	80 (41.7%)	57 (45.6%)
Recreational drug use (past 3 months)		
Yes	58 (29.6%)	26 (21.5%)
No	138 (70.1%)	95 (78.5%)
Time diagnosed with HIV		
0–5 years	32 (18.1%)	
5–10 years	32 (18.1%)	
10–20 years	61 (34.5%)	
20 + years	52 (2.4%)	
Currently on ART		
Yes	171 (94.5%)	
No	24 (5.5%)	

^a^p < 0.001 Using Chi squared test for HIV-positive versus HIV-negative groups

Of the HIV positive participants, 32 (18.1%) had been diagnosed with HIV for 0–5 years, 32 (18.1%) for 5–10 years, 61 (34.5%) for 10–20 years and 52 (29.4%) for more than 20 years. The majority were currently on ART (N = 171, 94.5%). 89% of all PWH, and 93% of those using ART, had an undetectable plasma HIV load (< 40 HIV-1 RNA copies/mL) at their last clinic visit.

There was a higher proportion of MSM in the HIV-positive compared to HIV negative group (66.5% vs. 24.2%; p < 0.001), and fewer heterosexual men (13.7% vs. 46.8%) and women (19.8% vs. 29.0%). PWH were more likely to be Black African (16.3% vs. 6.3%) than HIV negative participants, but there was no significant difference in ethnicity. No difference was observed in terms of levels of education, with similar proportions of HIV-positive and negative participants attaining a university qualification (54.4% vs. 60.7%) and having no qualification (12.3% vs. 11.5%). However, PWH were less likely to be employed (58.1% vs. 81.6%), and more likely to be retired (9.2% vs. 3.2%) or not working due to sickness/disability (14.2% vs. 4.0%) than HIV-negative participants (p < 0.001). Despite this there was no substantial difference in the number of participants who always had money for basic needs (57.9% vs. 66.4%).

There were no significant differences between the HIV-positive and HIV-negative groups in relation to recreational drug use (29.6% vs. 21.5% respectively) or proportions who were current smokers (30.1% vs. 36.0%), ex-smokers (28.1% vs. 18.4%) or non-smokers (41.7% vs. 45.6%). These characteristics are shown in Table 1.

### Resilience

Within the entire study population (HIV-positive and HIV-negative, N = 321), the mean resilience score was 76.9 (SD = 17.7); corresponding to a rating of ‘moderate’ resilience. The range was 14–98; the entire range of the RS-14 scale. The mean RS-14 score was 76 (SD = 18.1) for HIV positive participants and 78.5 (SD = 16.5) for HIV negative participants, for which there was no significant difference (t = 1.25 (df = 319), p = 0.2). Resilience did not differ significantly by HIV status when adjusted for age, gender/sexual orientation, and country of birth (adjusted Odds Ratio of high resilience, HIV positive reference group = 1.51, 95% CI 0.68–1.95, p = 0.61).

Table 2 reports the prevalence of resilience categorised by HIV status and age group. In unadjusted analysis, age group was not significantly associated with high resilience in either the HIV positive [χ^2^ test for trend, (N = 195) = 4.93, p = 0.55] or HIV negative groups [χ^2^, (N = 126) = 0.96, p = 0.99]. The adjusted association of age with high resilience is presented in Table 3, for HIV positive and HIV-negative participants separately. Among HIV-positive participants (after adjustment for gender/sexuality, time diagnosed with HIV and country of birth) the proportion of subjects with high resilience tended to increase with older age (test for trend Odds ratio 1.52, CI 0.95–2.41, p = 0.08). Longer time diagnosed with HIV, on the other hand, was related to a decrease in the proportion with high resilience (test for trend Odds ratio 0.37, CI 0.14–0.98, p = 0.05). Among HIV-negative participants, there was no association between age and resilience. Country of birth and gender/sexual orientation were not significantly associated with resilience in either group.

**Table 2:** Distribution of resilience score (RS-14 groupings) by age group and HIV status N (%)

Age in years	HIV positive (N = 195)			HIV negative (N = 126)		
	Low (≤ 64)	Average (65–81)	High (≥ 82)	Low (≤ 64)	Average (65–81)	High (≥ 82)
< 40 (N = 108)	20 (50.0%)	6 (15.0%)	14 (35.0%)	20 (29.4%)	16 (23.5%)	32 (47.1%)
40–50 (N = 103)	24 (33.3%)	11 (15.3%)	37 (51.4%)	7 (22.6%)	9 (29.0%)	15 (48.4%)
≥ 50 (N = 110)	29 (34.9%)	16 (19.3%)	38 (45.8%)	7 (25.9%)	6 (22.2%)	14 (51.9%)

Chi squared test for trend showed no significant difference (p = 0.2)

**Table 3:** Adjusted association of age, time with diagnosed HIV, gender/sexuality and country of birth with high resilience in HIV positive and negative adults (logistic regression analyses)

Independent Variable	High resilience (score ≥ 82)			High resilience (score ≥ 82)		
	p value	Odds ratio^a^	95% CI	p value	Odds ratio^a^	95% CI
Age	0.11			0.78		
Test for trend^b^	0.08	1.52	(0.95, 2.41)	0.48	1.18	(0.75, 1.87)
< 40	0.05	0.37	(0.14, 0.98)	0.48	0.71	(0.28, 1.82)
40–50	0.82	0.92	(0.45, 1.88)	0.68	0.80	(0.27, 2.32)
50 + ^c^		1			1	
Years with diagnosed HIV	0.22					
Test for trendb	0.05	0.37	(0.14, 0.98)			
0–5^c^		1				
5–10	0.89	0.93	(0.32, 2.65)			
10–20	0.36	0.65	(0.26, 1.65)			
20 +	0.07	0.38	(0.13, 1.08)			
Gender/sexual orientation	0.64			0.54		
MSM^c^		1			1	
Hetero male	0.51	1.39	(0.51, 3.79)	0.30	1.64	(0.65, 4.13)
Female	0.41	1.41	(0.63, 3.15)	0.35	1.63	(0.59, 4.57)
Country of birth						
Other^c^		1			1	
UK	0.92	0.96	(0.49, 1.89)	0.26	0.65	(0.31, 1.37)

^a^Multivariable logistic regression models with high resilience (score ≥ 82) as the dependent variable and including all factors in table as independent variables. Separate models for HIV+ ve and HIV− ve groups. p‐values obtained using likelihood ratio tests

^b^Linear trend across groups

^c^Reference group

*CI* confidence intervals, *MSM* men who have sex with men

### The Relationship Between Resilience and Mental and Physical Health

The presence of depression was reported by 39 (19.8%) HIV positive participants and 17 (13.1%) HIV negative participants. Anxiety was reported by 32 (16.2%) and 9 (6.9%) respectively and problems with ADLs by 69 (35.0%) and 21 (16.2%) respectively. Compared to HIV-negative participants, HIV-positive participants were significantly more likely to show the presence of anxiety [χ^2^, (N = 327) = 6.2, p = 0.009] and problems with ADLs [χ^2^, (N = 327) = 13.98, p < 0.001], but not depression [χ^2^, (N = 327) = 2.5, p = 0.07].

The unadjusted associations of resilience with depression, anxiety, and ADL problems were assessed for adults with and without HIV, and are shown in Table 4. In PWH, high levels of resilience were found to be significantly related to lower prevalence of depression (6.7% vs. 36.5% for prevalence of depressive symptoms among participants with high vs. moderate - low resilience), anxiety (3.4% vs. 29.7% respectively), and problems with ADLs (19.1% vs. 52.7% respectively; ps < 0.001, Chi squared tests). In adults without HIV, the overall prevalence of depression, anxiety and problems with ADLs were lower. However similar trends were seen in terms of the association between these measures and resilience, although in the HIV-negative group this was statistically significant only for depression (7.8% vs. 25.7%; for depression prevalence for high vs. moderate-low resilience p = 0.02).

**Table 4:** Physical and psychological symptom prevalence according to resilience level in adults with and without HIV

	Resilience in adults with HIV			
	Low (score ≤ 73)	Average (74–81)	High (score ≥ 82)	p value
	(N = 74)	(N = 34)	(N = 89)	
Depression present^a^	27 (36.5%)	6 (17.6%)	6 (6.7%)	p < 0.001
Anxiety present^b^	22 (29.7%)	7 (20.6%)	3 (3.4%)	p < 0.001
ADL problems^c^	39 (52.7%)	13 (38.2%)	17 (19.1%)	p < 0.001

	Resilience in adults without HIV			
	Low (score ≤ 73)	Average (74–81)	High (score ≥ 82)	p value
	(N = 35)	(N = 27)	(N = 64)	
Depression present^a^	9 (25.7%)	3 (9.7%)	5 (7.8%)	p = 0.03
Anxiety present^b^	4 (11.4%)	2 (6.5%)	3 (4.7%)	p = 0.45
ADL problems^c^	9 (25.7%)	5 (16.1%)	7 (10.9%)	p = 0.16

p Values by Chi squared test for trend

RS-14 score ≥ 82

^a^PHQ-9 score ≥ 10

^b^GAD-7 score ≥ 10

^c^Identified 1 or more problem on the first three questions of the EURO-QOL-5D

Adjusted associations of age, gender/sexual orientation, country of birth, and time with diagnosed HIV with depression and anxiety symptoms are shown in Table 5 among HIV positive participants only. In adjusted analysis, in the first stage model which did not include resilience, there was evidence of trends towards lower depression (test for trend Odds ratio 0.6, CI 0.33–1.07, p = 0.08) and anxiety (test for trend Odds Ratio 0.51, CI 0.26–0.99, p = 0.05) with older age in HIV positive participants. Time diagnosed with HIV was not significantly related to depression in this sample, although there was a trend of higher prevalence of anxiety with longer time with diagnosed HIV (test for trend Odds Ratio 1.6, CI 0.98–2.61, p = 0.06). In the second stage of analysis, when resilience was included as an independent variable, the associations of younger age with depression and anxiety symptoms among the HIV-positive group were attenuated (test for trend Odds Ratios 0.72, CI 0.39–1.33, p = 0.29 and test for trend Odds Ratio 0.59, CI 0.28–1.20, p = 0.14 respectively). Similarly the association of time with diagnosed HIV with anxiety was attenuated when resilience was included in the model. (test for trend Odds Ratio 1.54, CI 0.90–2.66, p = 0.12). However, resilience was strongly and independently inversely associated with both depression and anxiety symptoms in the adjusted models (test for trend Odds Ratios 0.37, CI 0.23–0.62, p < 0.001 and test for trend Odds Ratio 0.35, CI 0.20–0.61, p < 0.001 respectively).

**Table 5:** Adjusted association of age, time with diagnosed HIV, gender/sexuality and country of birth with depression, anxiety, and problems with ADLs in HIV-positive adults (logistic regression analysis)

(N = 197)	Depression (PHQ-9 score ≥ 10)						Anxiety (GAD-7 score ≥ 10)						One or more problems with ADLs					
	Step 1			Step 2			Step 1			Step 2			Step 1			Step 2		
Independent variable	p value	Odds ratio^a^	95% CI	p value	Odds ratio^a^	95% CI	p value	Odds ratio^a^	95% CI	p value	Odds ratio^a^	95% CI	p value	Odds ratio^a^	95% CI	p value	Odds ratio^a^	95% CI
Age	0.21			0.39			0.11			0.15			0.72			0.72		
Test for trend^b^	0.08	0.60	(0.33, 1.07)	0.29	0.72	(0.39, 1.33)	0.05	0.51	(0.26, 0.99)	0.14	0.59	(0.28, 1.20)	0.88	0.96	(0.57, 1.61)	0.68	1.12	(0.65, 1.95)
< 40	0.10	2.70	(0.82, 8.94)	0.39	1.75	(0.48, 6.32)	0.09	3.40	(0.83, 13.94)	0.29	2.26	(0.49, 10.41)	0.67	1.27	(0.43, 3.78)	0.89	0.92	(0.29, 2.96)
40–50	0.17	1.90	(0.75, 4.82)	0.18	1.99	(0.74, 5.39)	0.05	2.72	(0.98, 7.49)	0.05	2.99	(0.99, 9.03)	0.63	0.82	(0.38, 1.79)	0.43	0.71	(0.31, 1.65)
50 +^c^		1			1			1			1			1			1	
Years with diagnosed HIV	0.70			0.96			0.06			0.15			0.01			0.03		
Test for trend^b^	0.35	1.23	(0.80, 1.87)	0.67	1.11	(0.70, 1.75)	0.06	1.60	(0.98, 2.61)	0.12	1.54	(0.90, 2.66)	0.001	2.03	(1.35, 3.03)	0.003	1.93	(1.25, 2.98)
0–5^c^		1			1			1			1			1			1	
5–10	0.69	1.30	(0.36, 4.76)	0.95	1.04	(0.26, 4.27)	0.45	1.75	(0.40, 7.58)	0.81	1.22	(0.24, 6.24)	0.02	5.51	(1.29, 23.56)	0.04	5.05	(1.09, 23.52)
10–20	0.77	1.20	(0.37, 3.90)	0.96	1.03	(0.29, 3.73)	0.89	1.11	(0.28, 4.45)	0.91	0.92	(0.20, 4.23)	0.02	5.45	(1.37, 21.62)	0.02	6.16	(1.40, 27.12)
20 +	0.30	2.02	(0.54, 7.51)	0.66	1.38	(0.34, 5.61)	0.04	4.70	(1.09, 20.26)	0.12	3.54	(0.73, 17.12)	<.001	13.76	(3.16, 59.94)	0.002	11.00	(2.33, 51.98)
Resilience				0.001						0.002						0.001		
Test for trend^b^				<.001	0.37	(0.23, 0.62)				<.001	0.35	(0.20, 0.61)				<.001	0.47	(0.31, 0.70)
Low (< 73)^c^					1						1						1	
Average				0.11	0.39	(0.13, 1.22)				0.58	0.72	(0.23-2.27)				0.35	0.62	(0.22, 1.71)
High (> 82)				<.001	0.13	(0.05, 0.39)				<.001	0.10	(0.03, 0.35)				<.001	0.22	(0.10, 0.49)
Gender/sexuality	0.81			0.39			0.87			0.35			0.22			0.12		
MSM^c^		1			1			1			1			1			1	
Hetero male	0.53	1.46	(0.45, 4.77)	0.18	0.54	(0.22, 1.34)	0.92	1.08	(0.26, 4.50)	0.73	0.83	(0.29, 2.36)	0.09	2.61	(0.87, 7.78)	0.12	0.50	(0.21, 1.19)
Hetero female	0.73	1.19	(0.45, 3.17)	0.92	0.91	(0.17, 5.08)	0.63	0.75	(0.24, 2.36)	0.23	2.97	(0.50, 17.59)	0.47	1.39	(0.57, 3.40)	0.47	1.80	(0.36, 8.94)
Country of birth																		
Other^c^		1			1			1			1			1			1	
UK	0.65	1.21	(0.52, 2.80)	0.96	0.98	(0.40, 2.39)	0.12	2.19	(0.83, 5.80)	0.17	2.10	(0.74, 5.99)	0.03	2.45	(1.12, 5.35)	0.04	2.39	(1.03, 5.53)

Step 1 and 2 analysis

*CI* confidence intervals, *MSM* men who have sex with men

^a^Multivariable logistic regression model including all factors in table. p‐values obtained using likelihood ratio tests

^b^Linear trend across groups

^c^Reference group

No association was found between age and problems with ADLs (test for trend Odds Ratio 0.96, CI 0.57–1.61, p = 0.88) in PWH in adjusted analyses (Table 5). Longer time with HIV diagnosis was strongly associated with increasing ADL prevalence (test for trend Odds Ratio 2.03, CI 1.35–3.03, p = 0.001). With the addition of resilience to the analysis, the associations were similar (test for trend Odds Ratio 1.93, CI 1.25–2.98, p = 0.003). However, resilience itself was strongly inversely associated with problems with ADLs (test for trend Odds Ratio 0.47, CI 0.31–0.70, p < 0.001).

## Discussion

Comparable levels of ‘high’ resilience (48.4% vs. 45.6%) were found in adults with and without HIV respectively in this study. In adults with HIV, there was evidence that older age, and shorter time with diagnosed HIV were associated with ‘high’ levels of resilience. Older age and shorter time with diagnosed HIV also tended to be associated with lower prevalence of psychological symptoms (as was found in an earlier study of people with HIV [29]). High resilience was found to be significantly inversely related to the prevalence of depression, anxiety, and ADL problems in this population, and may, to some extent, mediate the relationship between age, time with diagnosed HIV, and mental health.

Resilience tended to increase with age, albeit nonsignificantly. While there is no literature available on age-related differences in resilience in PWH, resilience is thought to increase with age in the general population [7, 20, 23, 24, 39] in much the same way that well-being is found to increase from the age of 50 upwards [36]. In relation to resilience, this is possibly due to life experiences of coping with adversity [25]. Were this to be the true, one might expect to see a stronger association between resilience and age in PWH as they are likely to have experienced a greater number of life stressors. This may be apparent in our results—whereby an age-related association with resilience was suggested in the HIV positive population only. However, this data was under-powered to detect this, as the majority (54%) of HIV negative participants were under the age of 40. Despite this, in PWH the relationships of age and time diagnosed with HIV with depression and anxiety became weaker once resilience was included in the model. This gives some suggestion that high resilience may in part ‘explain’ why those who were older had lower prevalence of psychological symptoms. As resilience was also found to be related to better mental health, this may partially explain the results identified through the larger, multi-centre ASTRA study [29] which showed more favourable mental health with older age in UK PWH despite lower levels of physical health.

In contrast to age, longer time with diagnosed HIV was related to a decrease in the proportion of subjects with high resilience scores and an increase in problems with ADLs. Adults who have lived with HIV for prolonged periods of time, and especially through the period prior to development of combination ART (when HIV was viewed as a terminal illness), may have experienced a continued accumulation of stressors related to illness, stigma, and ageing with HIV, which could prevent them from developing resilience. In other words, similar to the toxic stress model [26], prolonged ill health and stress may inhibit the development of positive mental responses in PWH by restricting the resources necessary to cope with stress. This would result in low resilience in adults diagnosed with HIV for a long time, which could then exacerbate physical and mental stress. Resilience may therefore explain (at least in part) the higher levels of depression and anxiety reported in long-term diagnosed PWH [29] and resilience-based interventions could be considered within this sub-population in order to improve well-being. This may lead to improved outcomes as resilience has been related to medication adherence [31], and may well relate to engagement in care, both of which are important to the life-long treatment of complex chronic illnesses. Such interventions have been piloted amongst patients with diabetes [40], for example, with positive results.

Two previous studies have reported high levels of depression among people with HIV [29, 41], which was supported by the results presented here, but none have assessed the association of mental health with resilience in adults ageing with HIV. In the present study, high resilience was significantly related to lower prevalence of symptoms of depression and anxiety in PWH and lower prevalence of problems with ADLs. While no previous studies have explored the relationship between resilience and well-being in an HIV positive population, resilience has been consistently linked to mental [16, 20] and physical health [17–19] and health-care activities [22, 42, 43] in the general population. A possible reason for this is that resilient individuals are more ‘self-reliant’ [7, 8, 10] and perseverant [10] and may be more likely to engage in health-promoting behaviours such as healthy eating and exercise [43] which, in turn, improves overall mental and physical health. High levels of resilience may also buffer adults against mental distress by allowing for the development of adaptive coping mechanisms [4, 5, 44]. From the data presented here, it appears that high levels of resilience are related to improved mental and physical well-being in the HIV positive population.

One previous study has compared resilience by HIV status, and also reported somewhat lower levels of resilience in PWH compared to people without, but the comparison was not significant [30]. Our results are similar to these in that overall, HIV status was not significantly associated with resilience. It is possible, therefore, that HIV does not affect resilience in the same way as other chronic illnesses [19, 22]. This could be due to the large amount of support available to PWH through their HIV clinic [37, 38], which could enable positive mental well-being. However, the results relating to time diagnosed with HIV do not support this suggestion. Time with diagnosed HIV was inversely associated with high resilience, and those who have been diagnosed with HIV for longer would potentially have received more support [38], but showed lower levels of resilience. However, the limitations present in early HIV care and medication may have counteracted any such support. As both the previous [30] and current study utilised small sample sizes, it is also possible that there is a genuine difference in resilience scores according to HIV-status, but that a larger study is necessary in order to identify this difference. Previous studies have suggested that it is possible to develop or maintain resilience despite poor physical health [19, 21, 22]. Several studies have also suggested a causal relationship between resilience and well-being [18–20]. As resilience was not found to be significantly associated with HIV status, but was related to mental and physical health within our population, it may be possible to improve mental and physical well-being in adults with HIV through a resilience-based intervention.

## Limitations

There are limitations to this analysis. The HIV-negative group may have included a small number of individuals with undiagnosed HIV (those who had declined HIV-testing, or who tested positive subsequent to completion of the questionnaire). This may have impacted the mental and physical health scores reported in the HIV-negative group. However, the life experiences of those undiagnosed with HIV are likely to be different to those diagnosed and undergoing treatment. As such we consider them to be a distinct and separate group to PWH.

The sample size of older HIV-negative participants was small due to the lack of older adults attending the GUM clinic, which resulted in lack of power for age-related comparisons, as shown in the width of the confidence intervals reported. Due to the sample size we were required to combine all adults over the age of 50 into one group, preventing further analysis within the older adult population. The small sample size also limited the power of this study and may have resulted in type two errors.

The sample size also prevented us from performing sub-group analysis in relation to gender or ethnicity, or interaction tests. As significant differences were found in the proportion of participants by gender/sexual orientation between the HIV positive and negative groups, this may have impacted the HIV status group comparisons. However, as there was no significant association found between resilience and gender/sexual orientation, we do not consider this to be a major limitation.

It is also important to note that the associations reported are only correlational and, while causal explanations have been suggested, longitudinal data is required to provide stronger evidence of causality. As with any single-site study, the generalisability of these findings to other populations is currently unknown.

## Conclusions and Implications

This study provides the first data on resilience among PWH in the UK, and the associations of resilience with age and time with diagnosed HIV. It is the first to explore the relationship between mental and physical well-being with resilience in adults with HIV, and one of the first to explore resilience in comparison to HIV negative adults. The results suggest that, while resilience appears to increase with age, it appears to decline with increasing time diagnosed with HIV. As resilience was found to be inversely related to the prevalence of depression, anxiety, and physical health problems, it may mediate the associations of age and time with diagnosed HIV with mental health. Furthermore, resilience was not associated with HIV status overall, suggesting that it is possible to develop resilience within this population. Resilience and potential interventions to improve resilience are likely, therefore, to become increasingly important as the HIV positive population ages, and may have important implications for the care of HIV positive people in the future.
